# Risk of Bias in Reports of In Vivo Research: A Focus for Improvement

**DOI:** 10.1371/journal.pbio.1002273

**Published:** 2015-10-13

**Authors:** Malcolm R. Macleod, Aaron Lawson McLean, Aikaterini Kyriakopoulou, Stylianos Serghiou, Arno de Wilde, Nicki Sherratt, Theo Hirst, Rachel Hemblade, Zsanett Bahor, Cristina Nunes-Fonseca, Aparna Potluru, Andrew Thomson, Julija Baginskitae, Kieren Egan, Hanna Vesterinen, Gillian L. Currie, Leonid Churilov, David W. Howells, Emily S. Sena

**Affiliations:** 1 Centre for Clinical Brain Sciences, University of Edinburgh, Edinburgh, United Kingdom; 2 Medical School, University Medical Centre, Utrecht, Netherlands; 3 Statistics and Informatics Platform, Florey Institute of Neuroscience and Mental Health, Melbourne, Australia; 4 Faculty of Health, University of Tasmania, Hobart, Australia

## Abstract

The reliability of experimental findings depends on the rigour of experimental design. Here we show limited reporting of measures to reduce the risk of bias in a random sample of life sciences publications, significantly lower reporting of randomisation in work published in journals of high impact, and very limited reporting of measures to reduce the risk of bias in publications from leading United Kingdom institutions. Ascertainment of differences between institutions might serve both as a measure of research quality and as a tool for institutional efforts to improve research quality.

## Abstract 2

A systematic analysis of in vivo research reveals poor reporting of measures that reduce the risk of bias and an inverse relationship between impact factor and the reporting of randomization.

Bias occurs in in vivo research when there is a systematic error, or deviation from the truth, in the results of a study or the conclusions drawn from it. There are a large number of potential sources of bias, and the risks of some of the most important of these (selection bias and measurement bias) may be mitigated through simple study design features (randomisation and blinded assessment of outcome) [1,2]. Where risks of bias have been measured in systematic reviews of in vivo studies, a low prevalence of reporting of such measures has been found, and it is usual to find the largest reported efficacy in those studies meeting the lowest number of checklist items [3–7]. Improving the conduct and reporting of in vivo research is a stated priority for many funders and publishers [8–10].

Measuring the impact of scientific work is important for research funders, journals, institutions, and, not least, for researchers themselves. These measures are usually based on journal impact factor or citation counts and are used to inform individual and institutional funding decisions and academic promotions and to establish an informal hierarchy of journals that in turn guides where authors choose to submit their work. A journal’s impact factor is derived from the number of citations to articles published in that journal during the preceding 2 years and as such reflects the extent to which publications in that journal have influenced other work. The use of journal impact factors for the purposes described above rests on an implied assumption that highly cited articles, and by extension journals with a high impact factor, describe high-quality research.

Previous research has identified a low prevalence of reporting of measures to reduce the risk of bias for specific animal disease models [6,7,11–14], with studies thus identified as being at risk of bias tending to give higher estimates of treatment effects [3,4]. Kilkenny and colleagues showed that research in the United States and UK that was funded by public institutions (National Institutes of Health [NIH], Medical Research Council [MRC], Wellcome) had a low prevalence of reporting of measures to reduce the risk of bias [15], and Baker et al. showed that, even after the endorsement of the Animal Research: Reporting of In Vivo Experiments (ARRIVE) guidelines, reporting in *PLOS* journals (which have been one of the most enthusiastic proponents of the ARRIVE guidelines) remains low [16]. We have reported low prevalence of measures to reduce the risk of bias in a random sample of laboratory biomedical research [17]. However, since most previous studies have focused on the neurosciences, the reporting of measures to reduce the risk of bias across in vivo research as a whole is not known.

In the UK, the Research Excellence Framework (REF; www.ref.ac.uk) assesses the quality of research in higher education institutions. This is done with the stated purposes of (1) providing guidance for funding bodies to inform the selective allocation of research funding, (2) accountability for public investment, and (3) benchmarking information. What makes for high quality or excellence in research has to date been considered a combination of journal impact factor alongside qualitative judgement. For the 2014 REF, quality is considered to be a function of originality, significance, and rigour, with an intention to measure these against “international research quality standards”—although these standards have not been defined. While the REF guidelines were clear that assessments would not be based on journal impact factor, the number of citations to an article was admissible, and while they took quality to be “scientific rigour and excellence, with regard to design, method, execution and analysis,” it is not clear whether, and how, this was measured. Against this, many in UK science have felt that, as with previous research assessment exercises, journal impact factor was the dominant consideration when institutions decided which work, and which scientists, should be submitted to the 2014 round (http://www.guardian.co.uk/science/occams-corner/2012/nov/30/1).

It is plausible that journal impact factor may reflect originality and significance, but these dimensions are difficult to measure. Rigour, defined here as the use of experimental designs that reduce the risk of bias, can be estimated by determining whether manuscripts report the use of such measures. While the optimal design of each experiment may be specific to the hypothesis being tested, there are, for in vivo experiments at least, some widely applicable approaches that reduce the risk of bias.

These approaches include random allocation of animals to an experimental group (to reduce confounding), blinded assessment of outcome measures (to reduce detection bias), a statement of sample size calculation (to provide reassurance that studies were adequately powered and that repeated testing of accumulating data was not performed), and reporting of animals excluded from the analysis (to guard against attrition bias and the ad hoc exclusion of data). Investigator conflict of interest might increase or decrease the risk of bias [18–20], and a statement of whether or not a conflict of interest exists may help the reader to judge whether this may have occurred. Concerns that much in vivo research appears not to report such measures led to the development of standards for the conduct and reporting of in vivo research in specific disease areas [21–24] and across disease areas [1,25].

Objective judgement of the rigour of published work relies, of necessity, on information contained in that publication. It is entirely possible that work was conducted with the greatest rigour, but without a clear description of how bias was reduced, the reader cannot make such a judgement. Further, the overstatement of effects in studies that do not report measures to reduce the risk of bias suggests, firstly, that many do not take such measures and, secondly, that the true impact of bias may be even greater than has been observed.

Large effect sizes or unexpectedly “interesting” findings might lead to publication in a journal of high impact, while in fact those observations were due to the play of chance, to poor experimental design, or to selective reporting of statistically significant effects from a host of outcomes that were measured [26]. For instance, for gene association studies in psychiatry, Munafo et al. have shown that it is commonplace for the first (usually small and underpowered) study of the effect of a particular gene on the risks of developing depression to show large effects and to be published in a journal of high impact, with subsequent (larger, more challenging, and more time-consuming) studies showing much smaller effects yet being published in journals of much lower impact [27].

To provide an overview of the reporting of measures to reduce the risk of bias, we generated a random sample of 2,000 publications indexed in PubMED. Details of all methods used are given in the supplementary material (S1 Text), and all datasets are available in the Dryad repository: http://dx.doi.org/10.5061/dryad.cs3t8 [28]. For these studies, we ascertained the reporting of randomisation where this would be appropriate, of the blinded assessment of outcome, of a sample size calculation, and of whether the authors had a potential conflict of interest.

Excluding those not in English (339) or with subject matter related to chemistry or physics (114) left 1,547 publications, of which 814 reported primary research. 149 of these (18%) reported hypothesis testing experiments using live animals (S1 Fig), and full texts for all but three of these were retrieved. Twenty-seven publications reported randomisation (out of 134 in which this would have been appropriate; 20%). Four of 146 (3%) reported the blinded assessment of outcome, 15 of 146 (10%) reported a conflict of interest statement, and none of the 146 reported a sample size calculation. Reporting of randomisation increased from 9% in the first quintile of year of publication (1941–1978) to 33% in the last quintile (2008–2012), blinded assessment of outcome from 0% to 7%, and conflict of interest reporting from 3% to 40% (Fig 1).

**Fig 1:**
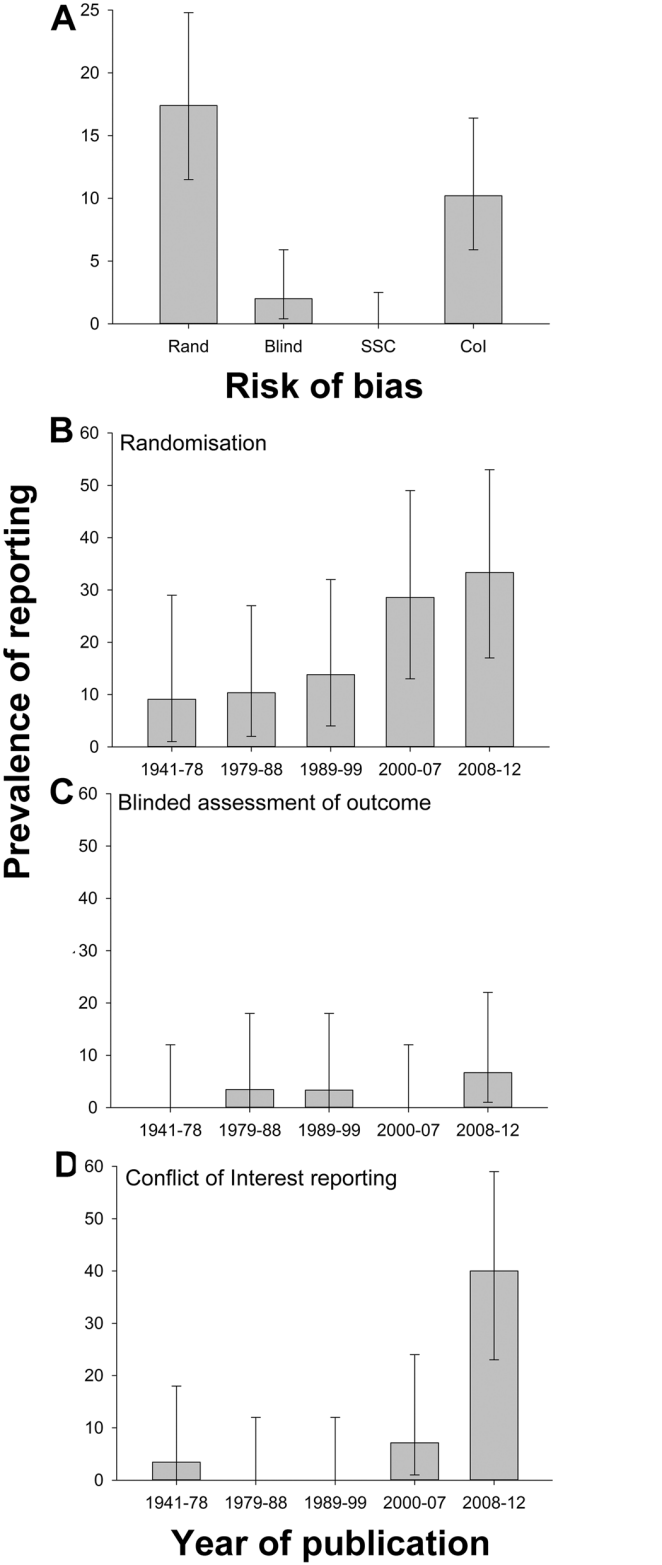
(A) Prevalence of reporting of randomisation, blinded assessment of outcome, sample size calculation, and conflict of interest in 146 publications describing in vivo research identified through random sampling from PubMed; change in prevalence of (B) randomisation, (C) blinded assessment of outcome, and (D) conflict of interest reporting in quintiles of year of publication. Vertical error bars represent the 95% confidence intervals of the estimates (S1 Data).

Next, we examined the reporting of measures to reduce the risk of bias in publications identified in a nonrandom sample of systematic reviews of in vivo studies. Since 2004, the Collaborative Approach to Meta-Analysis and Review of Experimental Data from Animal Studies (CAMARADES) has facilitated the conduct of systematic reviews and meta-analysis of data from in vivo experiments. Studies follow a common protocol [29] and approach to analysis [30]. Data from completed reviews, including the reporting of measures to reduce the risk of bias, are stored using common data architecture. Because we were also interested in any association between rigour and journal impact factor, we selected for this analysis those publications for which we could retrieve a journal impact factor for the year of publication.

We extracted data for 2,671 publications reporting drug efficacy in the eight disease models with highest representation in that dataset. Randomisation was reported in 662 publications (24.8%), blinded assessment of outcome in 788 (29.5%), a sample size calculation in 20 (0.7%), and a statement of potential conflict of interest in 308 (11.5%). There was substantial variation between different disease models in the prevalence of reporting of measures to reduce the risk of bias, being lowest for glioma and experimental autoimmune encephalomyelitis and highest for myocardial infarction (Fig 2). Reporting of randomisation increased from 14.0% (6/43) in 1992 to 42.0% in 2011 (31/77) (*p* < 0.001), reporting of the blinded assessment of outcome from 16.3% (7/43) to 39.0% (30/77) (*p* < 0.001), and reporting of a statement of possible conflict of interest from 2.3% (1/43) to 35.1% (27/77) (*p* < 0.001). The reporting of a sample size calculation did not change (2.3% [1/43] in 1992 and 1.3% [1/77] in 2011) (Fig 3).

**Fig 2:**
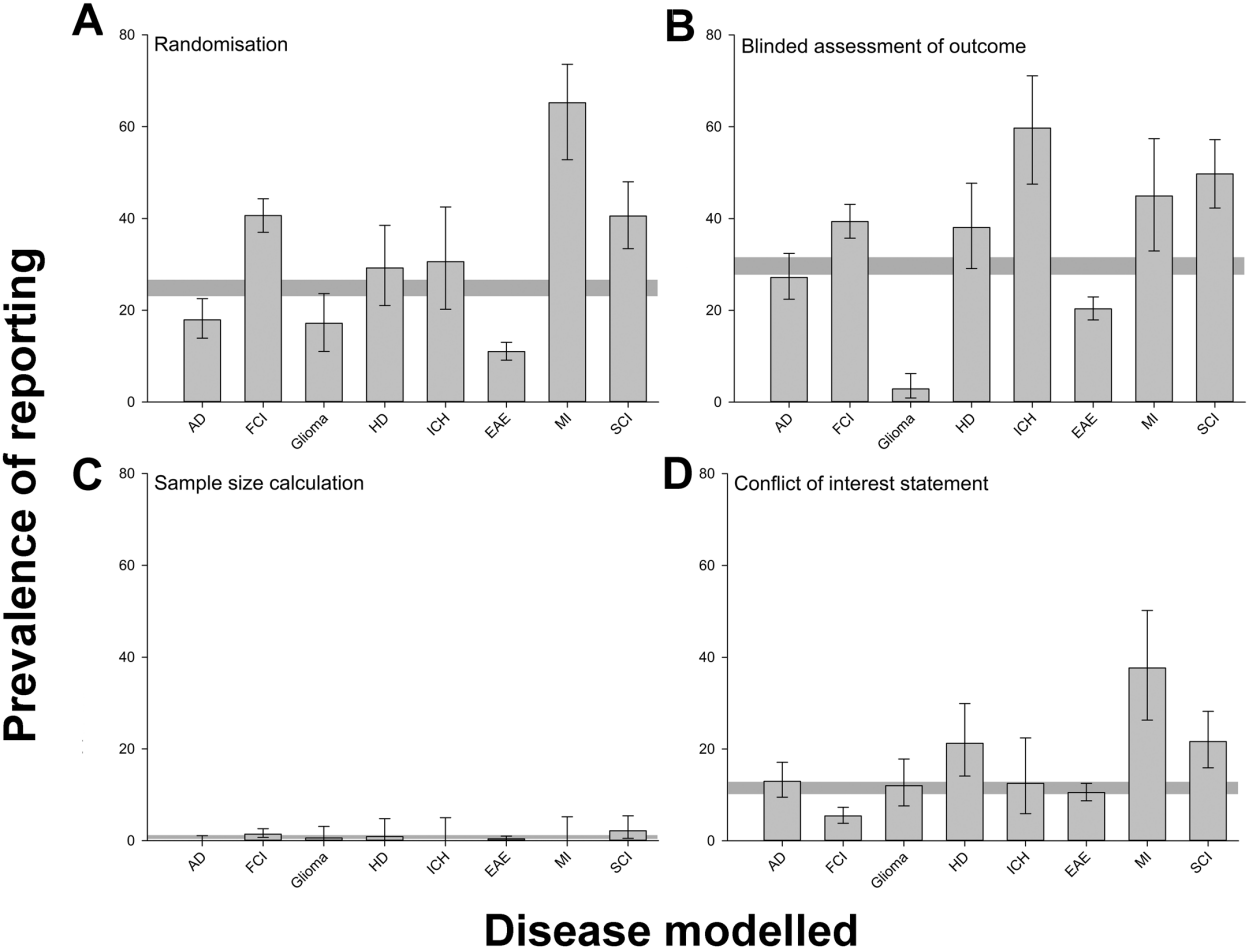
Prevalence of reporting of (A) randomisation, (B) blinded assessment of outcome, (C) sample size calculations, and (D) conflict of interest reporting in 2,671 publications describing the efficacy of interventions in animal models of Alzheimer’s disease (AD, *n* = 324 publications), focal cerebral ischaemia (FCI, 704), glioma (175), Huntington’s disease (HD, 113), intracerebral haemorrhage (ICH, 72), experimental autoimmune encephalomyelitis (EAE, 1029), myocardial infarction (MI, 69), and spinal cord injury (SCI, 185) identified in the context of systematic reviews. Vertical error bars represent the 95% confidence intervals, and the horizontal grey bar represents the 95% confidence interval of the overall estimate (S2 Data).

**Fig 3:**
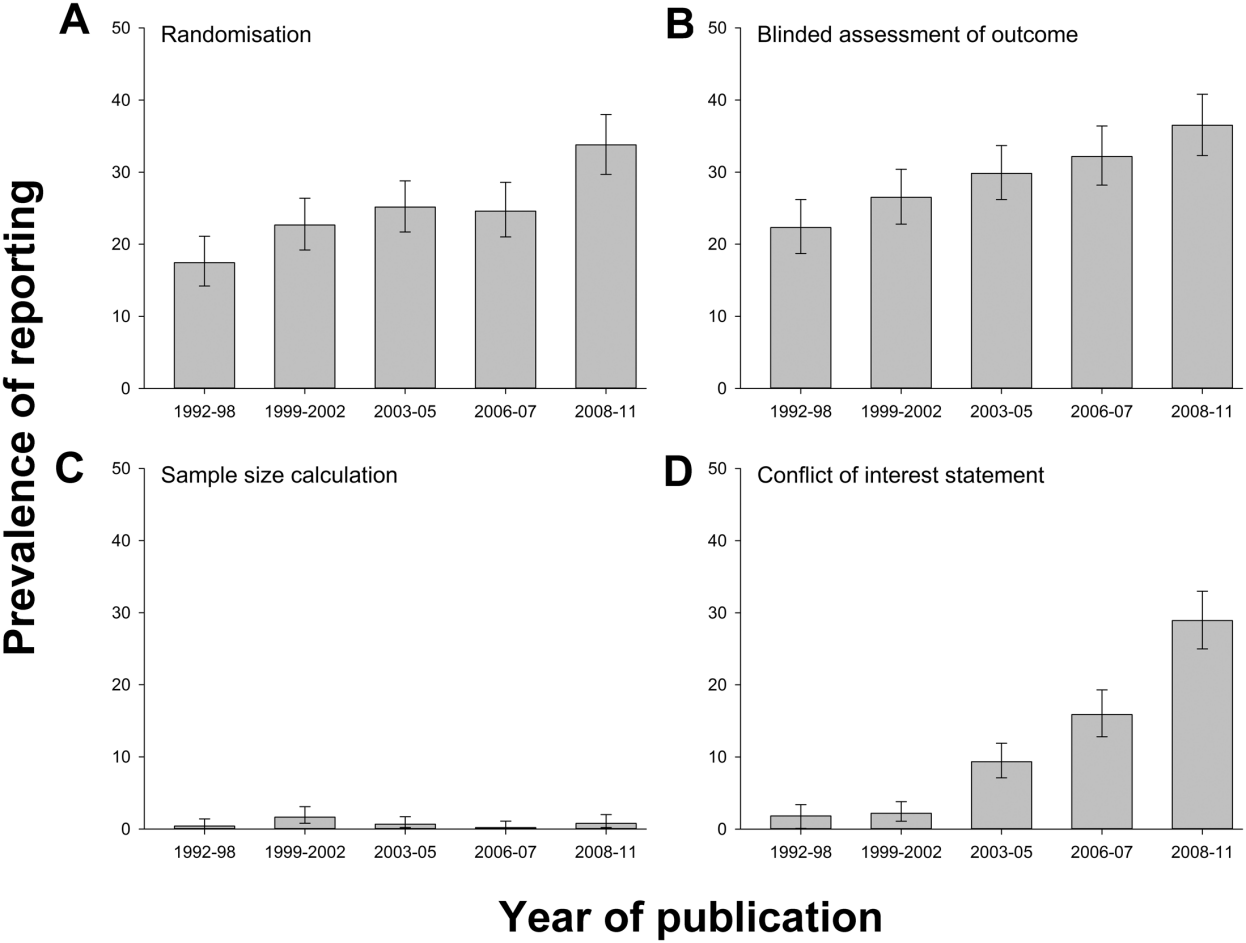
Change in prevalence of reporting of (A) randomisation, (B) blinded assessment of outcome, (C) sample size calculations, and (D) conflict of interest reporting in quintiles of year of publication for 2,671 publications describing the efficacy of interventions in animal models of eight different diseases identified in the context of systematic reviews. Vertical error bars represent the 95% confidence intervals of the estimates (S3 Data).

It is not known whether in vivo research published in high-impact journals is at lower risk of bias than that published in journals of lower impact. If scientists could be confident that their work would be judged on its own merits, then the case for expeditious publication in well-indexed online journals with open access, unlimited space, and the lowest publication costs would become unassailable. We therefore examined the relationship between journal impact factor and reporting of risks of bias in these 2,671 publications.

The median impact factor was 3.9 (interquartile range 2.6 to 6.3), and using median regression, there was no relationship between journal impact factor and the number of risk-of-bias items reported (beta coefficient 0.14, 95% CI −0.02–0.31, r = 0.024, *p* > 0.05). However, there were important differences in the reporting of individual risk-of-bias items. Median journal impact factor was 2.6 higher for studies reporting a potential conflict of interest (95% CI 2.4–2.9, r = 0.192, *p* < 0.001) but was 0.4 lower in studies reporting randomisation (95% CI 0.1–0.6, r = 0.047, *p* = 0.001). There was no significant difference for either the blinded assessment of outcome (−0.1, 95% CI −0.4–0.2, r = 0.056, *p* > 0.05) or sample size calculation (0.7, 95% CI −0.8–2.1, r = 0.000, *p* > 0.05).

The prevalence of reporting of measures to reduce the risk of bias in each decile of journal impact factor is shown in Fig 4. Only for a statement of a possible conflict of interest was reporting highest in the highest decile of impact factor, perhaps reflecting the editorial policies of such journals.

**Fig 4:**
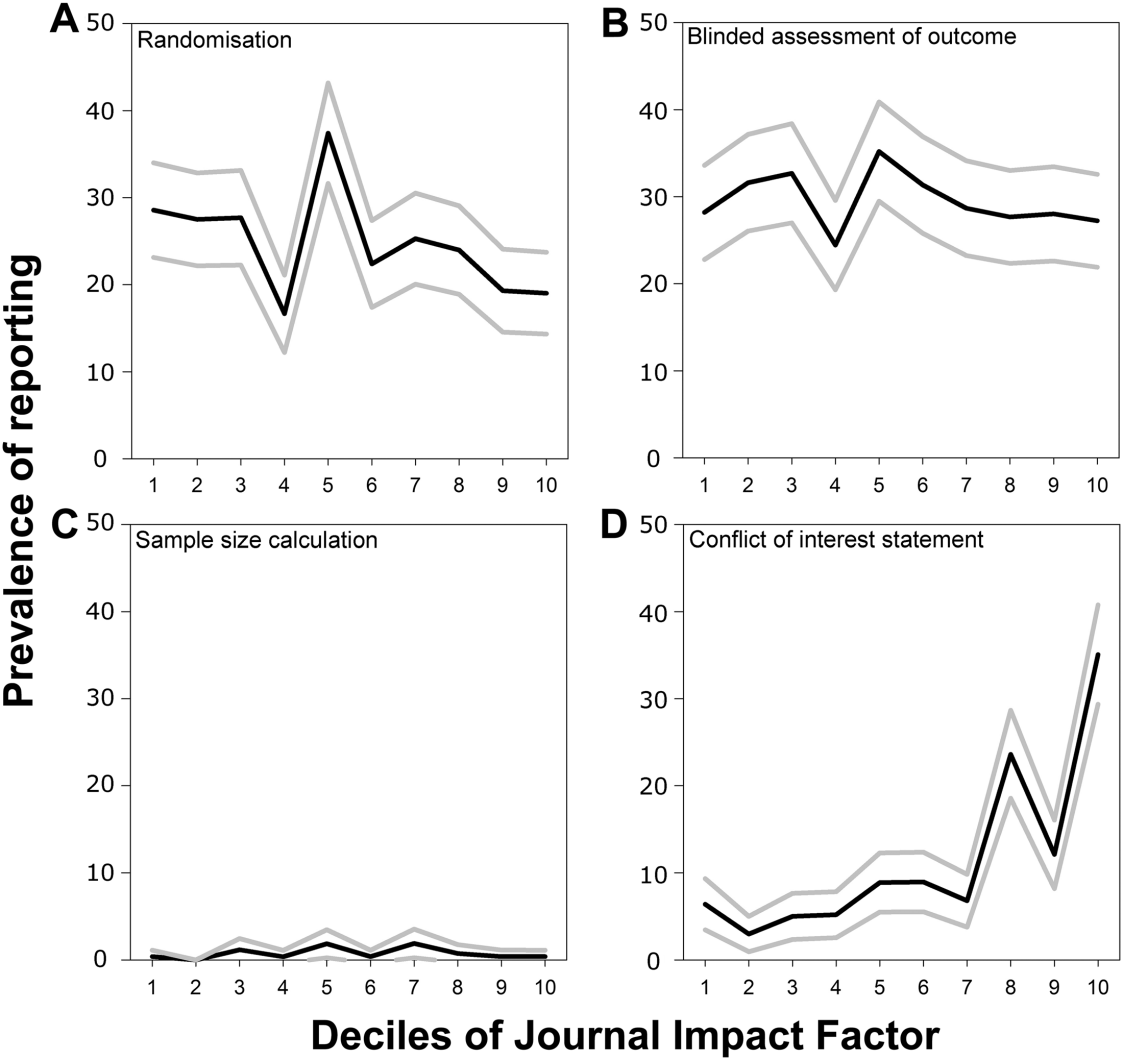
Prevalence of reporting of (A) randomisation, (B) blinded assessment of outcome, (C) sample size calculations, and (D) conflict of interest reporting by decile of journal impact factor in 2,671 publications describing the efficacy of interventions in animal models of eight different diseases identified in the context of systematic reviews. Black lines indicate the median value in that decile, and grey lines indicate the 95% confidence limits derived from nonparametric median regression (S4 Data).

We were also interested in whether the relationship between the reporting of measures to reduce the risk of bias and journal impact factor had changed over time. For the reporting of randomisation, there was such an interaction: in 1992 the median impact factor for studies reporting randomisation was 0.3 higher than for studies not reporting randomisation, but by 2011 this had reversed, with the median journal impact factor for studies reporting randomisation being 0.8 lower than that for studies not reporting randomisation.

Finally, we were interested to establish whether formal assessment of research performance is sensitive to these issues of rigour. To do this, we measured the reporting of measures to reduce the risk of bias in in vivo research published from the five UK institutions ranked highest across six units of assessment in biomedical sciences in the 2008 Research Assessment Exercise (RAE) (www.rae.ac.uk). We identified 4,859 publications from these institutions with a publication year of 2009 or 2010. By screening the title and abstract of these—and where necessary, the full text—we identified 1,173 publications that contained primary reports of in vivo research.

Alongside randomisation, blinding, and sample size estimation, Landis et al. [1] identified transparency of data handling, including the a priori determination of rules for inclusion and exclusion of subjects and data, as a core issue for study evaluation, For this analysis, we therefore assessed whether publications described such a priori determination, in place of ascertainment of the reporting of a potential conflict of interest.

Overall, 148 publications reported randomisation, of 1,028 in which this would have been appropriate (14.4%); 201 of 1,165 reported blinding (17.3%); 101 of 1,169 reported inclusion or exclusion criteria or both (10.4%); and 16 of 1,168 reported a sample size calculation (1.4%). Only one publication reported all four risk-of-bias measures [31], nine publications met three, 69 publications met two (7%), 297 publications met only one (32%), and 797 publications (68%) did not report any of the measures to reduce the risk of bias.

Further, there were interesting differences between institutions. The reporting of randomisation ranged from 7.2% (Institution B) to 16.3% (Institution A); the reporting of blinding from 12.4% (A) to 23.6% (C), the reporting of inclusion and exclusion criteria from 4.6% (D) to 11.6% (A), and the reporting of a sample size calculation from 0% (E) to 5.1% (D). There were significant differences between institutions in the reporting of each risk-of-bias item; for the reporting of randomisation, Institution B was significantly worse than all other institutions, and for the reporting of a sample size calculation, Institution D was significantly better than all other institutions (Fig 5). The rigour of research published from institutions judged more harshly by the RAE is a matter of the greatest interest, particularly given the consequences of such judgements on funding allocations.

**Fig 5:**
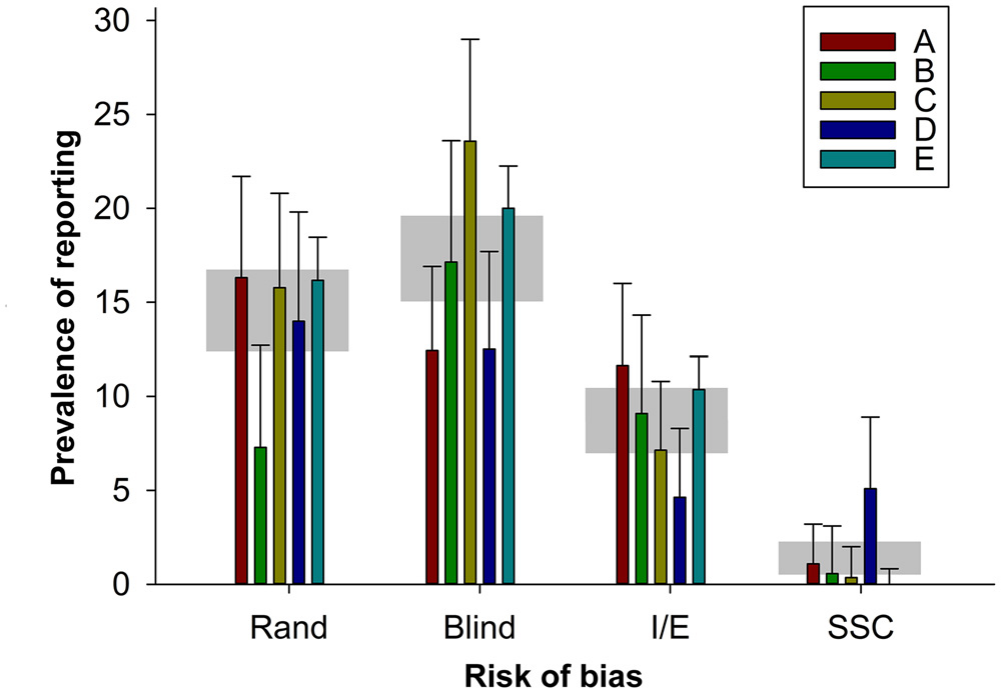
Prevalence of reporting of randomisation, blinded assessment of outcome, inclusion or exclusion criteria, and sample size calculation in 1,173 publications describing in vivo research published from five leading UK institutions (labelled A through E). For each institution, the vertical error bars represent the 95% confidence intervals, and the horizontal grey bar represents the 95% confidence interval of the overall estimate for that risk-of-bias item (S5 Data).

These datasets were assembled at different times for different purposes, and so comparisons between them have limited validity. However, it is possible to compare across the datasets the reporting of measures to reduce the risks of bias in those publications with a publication year of 2009 or 2010 (Table 1). Somewhat counter to expectations, reporting of randomisation was lowest (17.3%, 201/1165) in the RAE dataset, higher (35.7%, 76/213) in the CAMARADES dataset, and highest (50%, 7/14) in publications selected at random from PubMED.

**Table 1:** Reporting of measures to reduce the risk of bias in publications from 2009–2010 that were randomly selected, identified in the context of systematic reviews or from leading UK institutions.

	Randomisation		Blinding		Sample Size Calculation		
	*n*/*N*	% (95% CI)	*n*/*N*	% (95% CI)	*n*/*N*	% (95% CI)
PubMed	7/14	50 (23–77)	2/14	14 (2–43)	0/14	0 (0–23)
CAMARADES	76/213	36 (29–42)	79/213	37 (30–44)	2/213	1 (0–3)
Institutions	148/1028	14 (12–17)	201/1165	17 (15–20)	16/1168	1 (1–2)

There are of course a number of weaknesses to our approach. Foremost among these is that our measures of rigour (the reporting of measures to reduce the risk of bias) and of perceived quality (journal impact factor and RAE ranking) are of necessity very indirect: we were only able to ascertain whether publications reported measures to reduce the risk of bias, not whether they had actually done so. However, previous studies have shown that such reporting is associated with lower estimates of efficacy [3,4], and so it does appear that risk-of-bias reporting is, at worse, a surrogate measure of true risk of bias. Further, the importance of reporting methodological approaches in sufficient detail to allow replication is well established. For perceived study quality, the measures we used are relevant, being widely used to inform resource allocation decisions, including institutional funding and the promotion of individual scientists.

Secondly, we have not been able to consider the consequences of any upward drift of journal impact factor over time, although in the CAMARADES dataset, we observed only a small increase in mean impact factor, from 4.28 in 1992 to 4.40 in 2011.

Thirdly, we had a low threshold for considering studies to meet the various criteria. Bara et al. have shown [32] that for animal research carried in critical care journals, while some mention of randomisation was present in 47 of 77 publications (61%), the method of randomisation was described in only 1 (3%).

Finally, the relationship between institutional esteem and risk-of-bias reporting may be a consequence of the editorial practices of the journals in which high-quality research is reported; that is, they may be more likely to accept manuscripts from institutions of repute. Alternatively, the relationship between journal impact factor and risk of bias may be a consequence of such journals making publishing decisions based on institutional esteem rather than the quality of submitted manuscripts. However, the most parsimonious explanation of our findings is that journal editorial policies and those charged with assessing the quality of published work, including peer reviewers, have given insufficient attention to experimental design and the risk of bias, and that this has led investigators to believe that these factors are not as important as the novelty of their findings.

In spite of these weaknesses, we believe we have provided important empirical evidence of the reported rigour of biomedical research. Firstly, we show that reporting of measures to reduce the risk of bias in certain fields of research has increased over time, but there is still substantial room for improvement. Secondly, there appears to be little relationship between journal impact factor and reporting of risks of bias, consistent with previous claims that impact factor is a poor measure of research quality [33]. Thirdly, risk of bias was prevalent in a random sample of publications describing in vivo research. Finally, we found that recent publications from institutions identified in the UK 2008 RAE as producing research of the highest standards were in fact at substantial risk of bias, with less than a third reporting even one of four measures that might have improved the validity of their work. Further, there were significant differences between institutions in the reporting of such measures.

It is sobering that of over 1,000 publications from leading UK institutions, over two-thirds did not report even one of four items considered critical to reducing the risk of bias, and only one publication reported all four measures. A number of leading journals have taken steps that should over time improve the quality of the work they publish [8–10], and the effectiveness of the various different measures that have been taken will become clear over time. Such measures do not have to be expensive—Yordanov and colleagues have estimated [34] that half of 142 clinical trials at high risk of bias could be improved at low or no cost, and the same may well be true for animal experiments.

Of greater concern is that there still appears to be a lack of engagement with these issues amongst those charged with assessing the quality of published research. In the course of this work, we approached the UK Higher Education Funding Council (HEFC) seeking details of those publications submitted to them in the context of the 2014 REF; they declined a Freedom of Information request on the basis that disclosure would not, in their view, be in the public interest. The REF should of course have a role in championing good science, but it should also be a force for improvement, seeking to provide measures against which institutions and scientists can monitor improvements in the rigour of their research.

## Supporting Information

S1 Data Data underlying Fig 1. (XLSX)

S2 Data Data underlying Fig 2. (XLSX)

S3 Data Data underlying Fig 3. (XLSX)

S4 Data Data underlying Fig 4. (XLSX)

S5 Data Data underlying Fig 5. (XLSX)

S1 Fig Supplementary figure showing the fate of 2,000 publications selected at random. (TIF)

S1 Text Supplementary material including detailed methods. (DOCX)

## Abbreviations

ARRIVE: Animal Research: Reporting of In Vivo Experiments
CAMARADES: Collaborative Approach to Meta-Analysis and Review of Experimental Data from Animal Studies
HEFC: Higher Education Funding Council
MRC: Medical Research Council
RAE: Research Assessment Exercise
NIH: National Institutes of Health
REF: Research Excellence Framework
