# Correction: Risk of Bias in Reports of In Vivo Research: A Focus for Improvement

**DOI:** 10.1371/journal.pbio.1002301

**Published:** 2015-11-10

**Authors:** Malcolm R. Macleod, Aaron Lawson McLean, Aikaterini Kyriakopoulou, Stylianos Serghiou, Arno de Wilde, Nicki Sherratt, Theo Hirst, Rachel Hemblade, Zsanett Bahor, Cristina Nunes-Fonseca, Aparna Potluru, Andrew Thomson, Julija Baginskaite, Kieren Egan, Hanna Vesterinen, Gillian L. Currie, Leonid Churilov, David W. Howells, Emily S. Sena

The thirteenth author’s name is spelled incorrectly. The correct name is: Julija Baginskaite.

## References

[1] Macleod MR, Lawson McLean A, Kyriakopoulou A, Serghiou S, de Wilde A, Sherratt N, et al (2015) Risk of Bias in Reports of In Vivo Research: A Focus for Improvement. PLoS Biol 13(10): e1002273 doi: 10.1371/journal.pbio.1002273 26460723 10.1371/journal.pbio.1002273 PMC4603955

