# Supplementary figures and images for: Risk of Bias in Reports of In Vivo Research: A Focus for Improvement
Source: PLoS Biol. 2015 Oct 13;13(10):e1002273. doi: 10.1371/journal.pbio.1002273 (PMC4603955; doi:10.1371/journal.pbio.1002273)

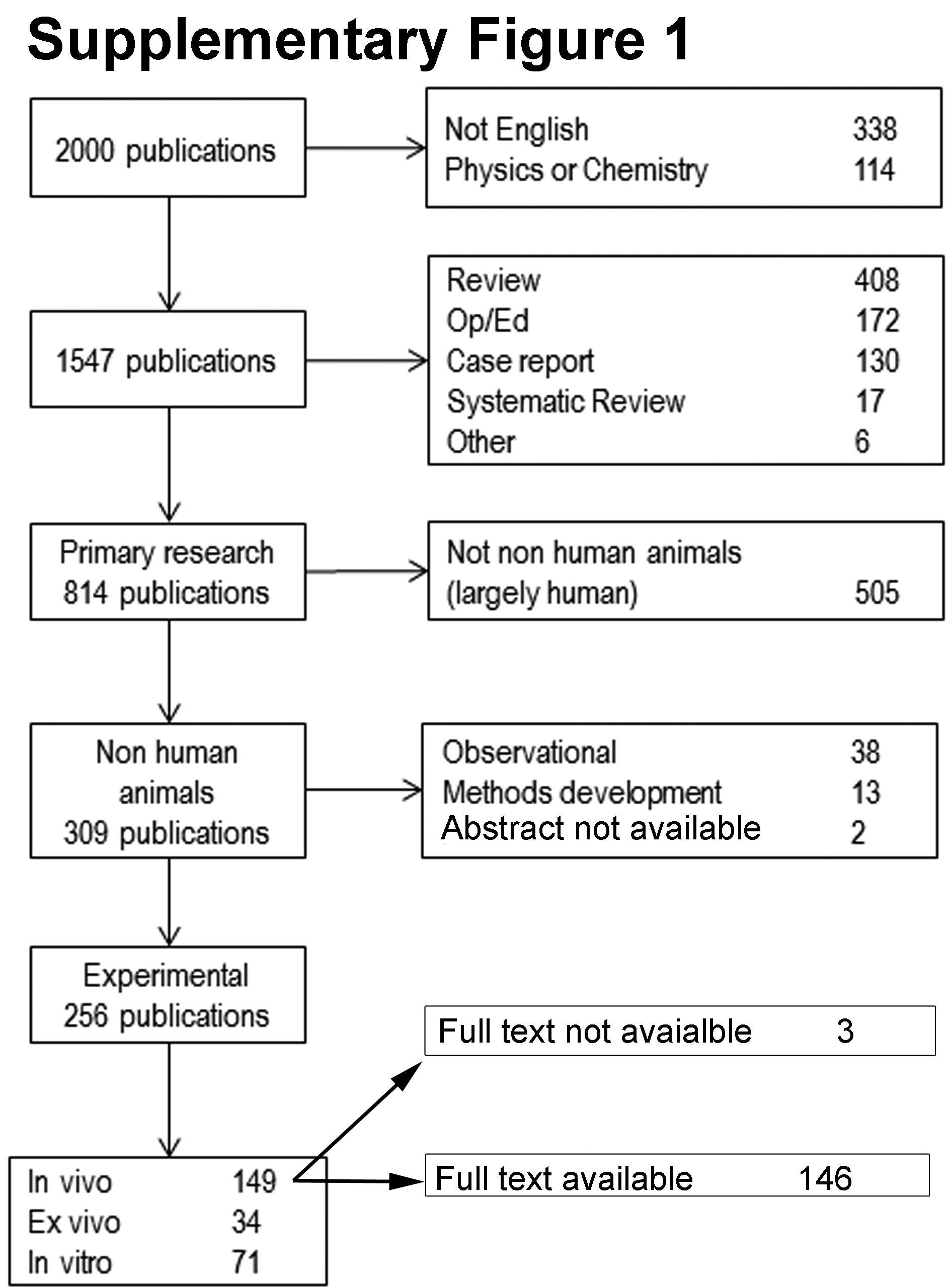

Supplement: S1 Fig — (TIF) [file pbio.1002273.s006.tif]
