# Supplementary material for: Risk of Bias in Reports of In Vivo Research: A Focus for Improvement
Source: PLoS Biol. 2015 Oct 13;13(10):e1002273. doi: 10.1371/journal.pbio.1002273 (PMC4603955; doi:10.1371/journal.pbio.1002273)
Supplement: S1 Text — (DOCX) [file pbio.1002273.s007.docx]

Supplementary Material

We conducted three separate analyses relating to the reporting of measures to reduce the risk of bias in *in vivo* research, defined here as the reporting of experiments involving non-human whole live animals as subjects. Where a juvenile or embryonic form was studied in its natural environment we considered this to be an *in vivo* experiment, whereas if it was removed to an artificial environment (eg *Xenopus* oocytes transfected and studied in a dish for neurophysiology) we did not.

Firstly we present data from a random sample from PubMed of publications reporting *in vivo* research. Next we present data collected in the context of CAMARADES systematic reviews (largely involving testing drug efficacy in animal models of neurological diseases), where we also explore any relationship of reporting of measures to reduce the risks of bias with the impact factor of the journal of publication. Finally, we present data from publications form 5 leading UK institutions, published in the two years following the 2008 Research Assessment Exercise where they were ranked as leading research institutions.

**Choice of risks of bias**

These datasets were initiated at different times (the earliest CAMARADES review was published in 2005), and the risks of bias which were ascertained differ slightly across the three studies. Each includes information for reporting of randomisation, blinded assessment of outcome and sample size calculation. In addition, the random Pubmed sample includes information for concealment of allocation sequence, blinded conduct of the experiment, and a statement regarding potential conflict of interest. From the CAMARADES dataset we have also extracted data for reporting of a potential conflict of interest, but data for concealment of allocation sequence and blinded conduct of the experiment had been inconsistently collected in contributing systematic reviews. At the time of initiation of data collection for publications from leading UK institutions we had been persuaded by Landis et al that reporting of criteria for inclusion, or of animals excluded from analysis, was more important than conflict of interest reporting, and so collected this instead. We did so in the basis that the process through which the Landis criteria were developed reflected something of a consensus in biomedical research at that time, and therefore would provide the most reasonable yardstick against which to consider contemporary research.

**Risk of bias in publications selected at random**

We generated 2000 random numbers between 1 and 23,000,000 using the “Rand()” command in Microsoft Excel. These were converted to a text format, and we used the find/replace function in Microsoft Word to add the string “[PMID] OR” between each number, to give a text string “rand_1_ [PMID] OR rand_2_ [PMID] OR … OR rand_n_ [PMID]”. This was then copied into the search field at [www.pubmed.com](http://www.pubmed.com/) and the search results retrieved as an xml file. Using Pubmed2XL (<http://blog.humaneguitarist.org/projects/pubmed2xl/>) this was converted to a Microsoft Excel file, which was then imported to Microsoft Access. We used an update query to add the term “[www.pubmed.com/](http://www.pubmed.com/)” before the PubMed ID, and converted this to a hyperlink to allow the relevant PubMed page for that article to be accessed from within Microsoft Access. We then designed a data entry form to allow relevant publication characteristics to be added to the database.

We selected publications that described *in vivo* research using non-human animals. In the first stage we determined whether a publication was a review article (including systematic reviews) or described primary research (including observational studies). We specifically excluded publications not in English, those exclusively in the fields of chemistry or physics, and descriptive reports, case reports, surveys, retrospective studies and correlation studies. We selected for further analysis publications describing experiments involving living biological non-human subjects as whole live animals.

For each publication the full text of the article was retrieved. For each study we recorded whether the publication reported a sample size calculation, randomisation, concealment of allocation sequence, blinded conduct of the experiment, blinded assessment of outcome, or a conflict of interest statement. We also recorded the species of origin of the biological material, the research design (experimental or observational) and the area of research. Where a publication described more than one *in vivo* experiment we scored each item at the highest level of reporting. For this and subsequent studies we considered that the reporting standard was met if there was any mention of for instance randomisation. We did not require the mechanism of randomisation to be described.

We scored reporting as being present, absent, or not applicable to the research design – for instance, where the phenotype of transgenic animals was being compared to wild type phenotype. Two observers independently scored risk of bias items, each blinded to the assessment of the other. Differences between the two observers were resolved by a third reviewer. The data collected were entered into the Collaborative Approach to Meta-Analysis and Review of Animal Data from Experimental Studies (CAMARADES) data manager application in Microsoft Access 2003.

For each risk of bias item we calculated a prevalence of reporting and 95% exact binomial confidence intervals (http://www.stata.com/manuals13/rci.pdf). We analysed change over time by considering studies in quintiles of year of publication and calculating prevalence in each quintile. We calculated the prevalence of reporting of each risk of bias item in each research domain.

From 2000 publications selected at random, we excluded 339 because they were in a language other than English and 114 because the subject matter related to chemistry or physics rather than biomedicine. Of 1547 remaining publications 814 reported primary research, of which 309 involved non-human animals or tissues from non-human animals. Of these 256 reported hypothesis testing experiments, of which 149 reported experiments using live animals (Supplementary figure 1). We were able to retrieve the full text for 146 of these. We compared this manual extraction with the PubMed tagging for animal experiments; 138 or 149 relevant studies, and 312 of 1851 non relevant studies were tagged as animal studies, giving sensitivity for the PubMed tagging of 93% and specificity of 83%.

**Journal impact factor and risk of bias**

We explored the relationship between the reporting of measures to reduce the risk of bias and the impact factor of the journal of publication for those *in vivo* models of disease represented in the CAMARADES database at 1^st^ September 2013 by at least 50 publications with an available impact factor. These publications had been identified in the context of systematic reviews addressing the efficacy of specified interventions in animal models of disease. We did not restrict inclusion by year of publication or by language. Investigators had, in the context of their systematic reviews, ascertained the reporting of a range of measures which might reduce the risk of bias; common to all reviews were (i) random allocation of subjects to experimental group, (ii) blinded assessment of outcome, (iii) a sample size calculation, and (iv) a statement regarding potential conflict of interest.

10 different *in vivo* disease models were represented by more than 50 publications. Of 3911 publications 521 had been published before 1992, the earliest year for which we could secure reliable impact factor data across the range of publications represented. A further 431 were published in journals that did not have a reported journal impact factor for the year of publication, and 247 publications were represented in the database more than once. Once these factors were taken into account, the number of publications describing Parkinson’s disease and lacunar stroke models had fallen below 50 and so were excluded from further analysis. For each publication we retrieved the Thomson Reuters impact factor for that journal for that year of publication.

The study protocol is available online at [www.camarades.info/protocols.html](http://www.camarades.info/protocols.html)/. We planned to use linear regression to examine the relationship between risk of bias items and journal impact factor. But because the residuals were not normally distributed (Shapiro-Wilk test z = 15.59, p<0.001), we instead used the non-parametric median regression approach (STATA command bsqreg, http://www.stata.com/help.cgi?bsqreg). Using the originally proposed approach of linear regression would also have shown a significant decline in the reporting of blinding as impact factor rose, so the effect of the change of analysis plan was to reduce rather than increase the number of significant findings. Because we were testing associations between 4 risk of bias items and journal impact factor we adjusted our critical value of p to 0.0125.

One important consideration is that if journal impact factors and the reporting of measures to reduce the risk of bias both increase over time, this would lead to an apparent positive association between journal impact factors and the reporting of measures to reduce the risk of bias over time; and equally, if one increased while the other declined this might obscure a negative relationship. However, there was no substantial change in journal impact factor for the publications included in this analysis, being 4.28 in 1992 and 4.40 in 2011.

**Risk of bias in publications from leading UK universities.**

We searched PubMed (September 2012) for publications with a Publication Year of 2009 or 2010 and with an institutional affiliation of the Universities of Oxford, Cambridge, Edinburgh, University College London or Imperial College London, chosen on the basis of their performance in the 2008 UK Research Assessment Exercise. Specifically, these had highest aggregate scores for the Units of Assessment of Cardiovascular Medicine, Cancer Studies, Infection and Immunity, Other hospital based clinical subjects, Psychiatry, neuroscience and clinical psychology, and Biological sciences. We identified experiments involving animals using the filter available in PubMed. We used the same criteria outlined above to identify primary reports of *in vivo* research involving non-human animals, and retrieved the full text publication for included studies. For this study we focussed on the risk of bias items recently identified [1] as being of particular importance, these being (i) random allocation of subjects to experimental group, (ii) blinded assessment of outcome, (iii) a sample size calculation, and (iv) a statement of criteria for inclusion or exclusion of animals or data from the experiment and from analysis. Where there were differences between experiments in the same paper in the reporting of measures to reduce the risk of bias, we scored the highest level of reporting. We selected these slightly different criteria as those identified by Landis et al[1] as being of particular salience, data collection for this part of the project commencing after publication of those criteria. Two observers independently scored risk of bias items, each blinded to the assessment of the other. Differences between the two observers were resolved by a third reviewer. Kappa statistics for agreement between the first two assessors was 0.66 for randomisation, 0.74 for blinding, 0.25 for inclusion and exclusion criteria and 0.35 for sample size calculations

It might be argued that failure to report a sample size calculation does not, in itself, constitute a risk of bias. We disagree. Firstly, where sample size is determined on the basis of a power calculation it makes it much less likely that incremental additions are made to group sizes until significance is “achieved”. This constitutes multiple statistical testing in the same population and while there are circumstances in which it can be justified, significance testing needs to be adjusted to account for it. Secondly, the power of a study is crucial to interpreting the findings in the context of what is already known. Where most studies are underpowered, the positive predictive value of a significant finding is reduced.[2] We have previously shown, in studies modelling multiple sclerosis, that reported effect size falls as group size (and therefore statistical power) increases.[3] We believe this may be due to publication bias; underpowered experiments will give less precise estimates of treatment effects, and where the 95% confidence interval for such estimates includes the null then the work is less likely to be published. Where larger effects are observed, these are less likely to include the null, and are more likely to be published.

For each risk of bias item we report an overall prevalence. To test the feasibility of using such metrics to compare institutions we report their prevalence in research from each institution, but because our purpose is not to report the performance of specific institutions we have labelled those institutions A through E.

**Data availability.**

The datasets for each component of the study are available at Dryad.

Reference List

(1) Landis SC, Amara SG, Asadullah K, Austin CP, Blumenstein R, Bradley EW, et al. A call for transparent reporting to optimize the predictive value of preclinical research. Nature 2012 Oct 11;490(7419):187-91.

(2) Ioannidis JP. Why most published research findings are false. PLoS Med 2005 Aug;2(8):e124.

(3) Vesterinen HM, Sena ES, ffrench-Constant C, Williams A, Chandran S, Macleod MR. Improving the translational hit of experimental treatments in multiple sclerosis. Mult Scler 2010 Sep;16(9):1044-55.
